# Bullous pemphigoid successfully treated with dupilumab^☆^

**DOI:** 10.1016/j.abd.2023.08.017

**Published:** 2024-06-13

**Authors:** Daniela de Abreu e Silva Martinez, Amanda de Freitas Sampaio Periquito, Graciela Galva Roa, João Pedro Lupi, Curt Mafra Treu, Omar Lupi

**Affiliations:** a Department of Immunology, Hospital Universitário Clementino Fraga Filho, Universidade Federal do Rio de Janeiro, Rio de Janeiro, RJ, Brazil; b Department of Dermatology, Policlínica Geral do Rio de Janeiro, Rio de Janeiro, RJ, Brazil; c Department of Medicine, Hospital Universitário Pedro Ernesto, Universidade Estadual do Rio de Janeiro, Rio de Janeiro, RJ, Brazil

*Dear Editor,*

Bullous pemphigoid (BP), a chronic autoimmune cutaneous blistering disorder affecting predominantly the elderly, is characterized by skin tense bullae formation and pruritus symptoms. At present, the main treatment options are represented by corticosteroids and immunosuppressant drugs. Steroids often need to be administered in high doses, with subsequent adverse events and safety issues.^1^, ^2^ Safer treatment modalities are therefore needed. Dupilumab is a biologic agent used to treat BP in recent years.^2^ Here, we describe an elderly patient with recalcitrant BP successfully treated with dupilumab.

An 89-year-old woman was admitted to our department with a 1-month history of severely itchy erythema, vesicles and blistering. On examination, there were urticarial plaques and vesicles on the trunk, and extremities. Isolated tense blisters could be observed on the abdomen and bilateral thighs (Fig. 1) with sparing of mucosal surfaces. While the lesions evolved into erosions (Fig. 2), the Nikolsky sign was negative. In addition, the patient had type 2 diabetes mellitus on metformin and hypothyroidism on levothyroxine for over 20 years, she denied taking any new medications. Laboratory investigations revealed mild eosinophilia; however, no other significant abnormalities were present. An extensive workup for underlying malignancy and rheumatologic diseases with imaging and laboratory test results proved unremarkable. Skin biopsy demonstrated subepidermal blister formation with eosinophilic infiltrate (Fig. 3). Direct immunofluorescence revealed subepidermal separation with continuous linear depositions of IgG and C3 along the basement membrane zone. The clinical picture in combination with the histologic and immunologic findings confirmed the diagnosis of BP. She was started on prednisone 80 mg, and after 2 flares during tapering of prednisone, the decision was made to proceed with azathioprine 200 mg, and tetracycline 500 mg. Although moderate doses of prednisone effectively reduced the severity of blistering lesions, her pruritus and diabetes were not sufficiently controlled. Attempts to very slowly taper her prednisone resulted in disease flares and severe pruritus itching over three years. Given the patient’s severe disease status and treatment limitations, her treatment was transitioned to a therapeutic trial of dupilumab, with an initial loading dose of 600 mg administered subcutaneously followed by weekly 300 mg subcutaneous injections. At the 6-month follow-up, there was a complete resolution of bullae and pruritus after treatment with dupilumab alone (Fig. 4).

**Figure 1:**
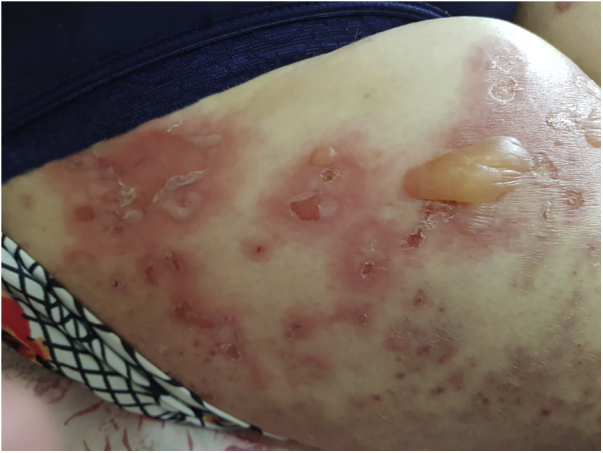
Isolated tight blisters can be seen on the right thigh.

**Figure 2:**
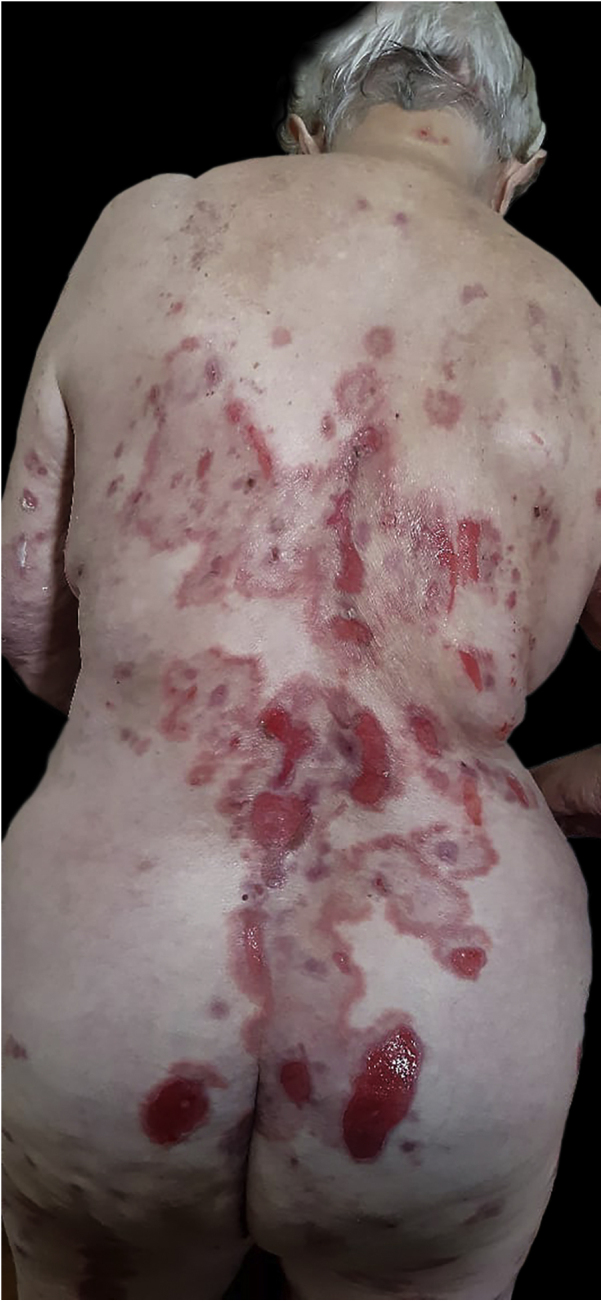
Erosions on the trunk and buttocks.

**Figure 3:**
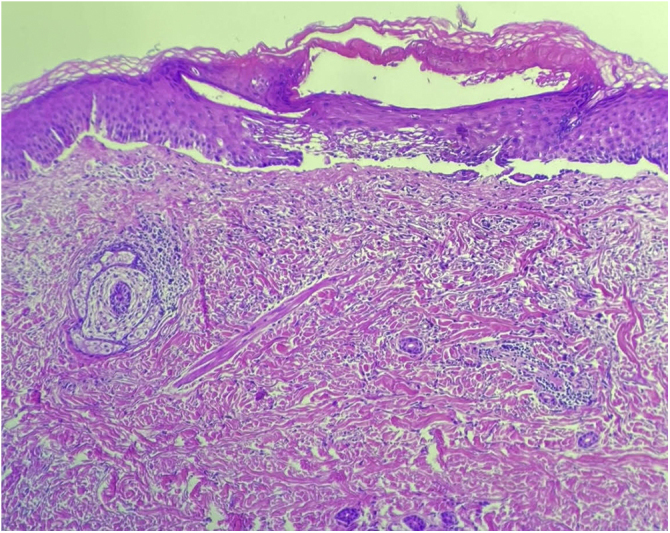
Hematoxylin & eosin, 100x. Revealing subepidermal blister formation with eosinophilic infiltrate.

**Figure 4:**
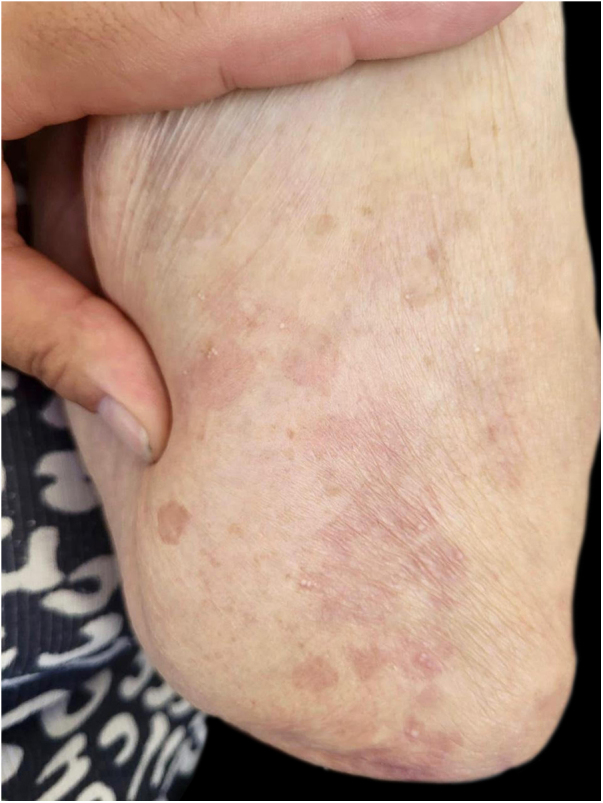
Lesions resolved with post-inflammatory hyperpigmantation.

BP is an autoimmune disease that mainly occurs in the elderly, severely affecting their health and quality of life.^3^ Treatment often entails topical/systemic corticosteroids, antibiotics, azathioprine, dapsone, methotrexate, and mycophenolate mofetil.^4^, ^5^ These regimens carry significant adverse effects, and careful consideration is warranted in elderly patients.

Type 2 inflammation is an immune response that exerts an important role in host defense against parasites and is predominantly mediated by group 2 Innate Lymphoid Cells (ILC2s), type 2 T-helper (Th2) cells, eosinophils, and relevant cytokines, such as IL-4, IL-5, and IL-13. A large number of stimuli can trigger type 2 inflammation, including helminths, various allergies, certain viral or bacterial infections, and endogenous molecules. The process involves both innate and adaptive immune responses.^1^, ^3^ An increasing number of studies revealed that BP is a Th2-dominant disease with subsequent overexpression of Th2-type cytokines such as IL4, IL5, and IL13. IL4 is specifically associated with the recruitment of eosinophils which contribute to the incessant pruritis present with the disease.^6^

Dupilumab is a recently developed monoclonal antibody that blocks the signaling of IL-4 and IL-13, both of which are crucial cytokines in the T2 response.^7^, ^8^, ^9^ For this reason, the hypothesis is that the reduction in disease activity obtained in the cases reported so far may be related to the reduction in Th2-type responses induced by the inhibition of IL-4 and IL-13 signal transduction induced by dupilumab.^1^ The European Academy of Dermatology and Venereology already considers dupilumab as an optional treatment for refractory BP.^10^

Treatment of BP can be challenging, especially in refractory cases. However, the role and efficacy of Dupilumab in the treatment of BP is not completely clear yet; Certainly, Dupilumab should not be considered the first-choice treatment, but as a rescue therapy for selected patients with recalcitrant BP. While the present case highlights the use of dupilumab as a novel therapy in the treatment of BP, additional studies are needed. The mild side effect profile of dupilumab would make it an ideal option for treating the elderly and patients with comorbidities.

## Financial support

None declared.

## Authors’ contributions

Daniela de Abreu e Silva Martinez: Preparation and writing of the manuscript; intellectual participation in propaedeutic and/or therapeutic management of studied cases; approval of the final version of the manuscript; critical literature review.

Amanda de Freitas Sampaio Periquito: Preparation and writing of the manuscript; approval of the final version of the manuscript.

Graciela Galva Roa: Preparation and writing of the manuscript; approval of the final version of the manuscript.

João Pedro Lupi: Preparation and writing of the manuscript; approval of the final version of the manuscript.

Curt Mafra Treu: Preparation and writing of the manuscript; intellectual participation in propaedeutic and/or therapeutic management of studied cases; approval of the final version of the manuscript.

Omar Lupi: Preparation and writing of the manuscript; intellectual participation in propaedeutic and/or therapeutic management of studied cases; approval of the final version of the manuscript.

## Conflicts of interest

None declared.
